# Correspondence

**Published:** 1896-02

**Authors:** George Horine

**Affiliations:** Americus, Ga.


### Correspondence.

*Editor* SOUTHERN MEDICAL RECORD:

With your kind permission I will contribute my mite to the rapidly increasing statistics in regard to the use of diphtheria antitoxine. It is the duty of every one to assist in establishing the truth, and while there are so many reports of success and of failure that it is quite bewildering to read them; still it is the sum total of the individual experience that must ultimately establish the truth. Of course, every one is familiar with the literature of antitoxine, and I shall only give my experience, limited though it is, in regard to its use in that much dreaded disease, diphtheria, and its influence on the mortality of the disease. In the past four years there have been nineteen well-marked cases of diphtheria in the city of Americus. Nine cases occurred prior to the introduction of the diphtheria antitoxine, and of these nine cases, five died, constituting a mortality of fifty-five per centum. They were treated locally and constitutionally, according to the best methods then known to the profession. Of the five fatal cases, four died of the primary trouble and one of the paralysis that follows as a sequel to diphtheria.

Of the ten cases treated with the antitoxine all recovered, although one patient died in the family where three recovered under antitoxine. The one that died was not treated with antitoxine, because we could not obtain it in time; but no death occurred after we began using antitoxine. No bad effects of any kind followed the use of the antitoxine. It has been claimed by some that nephritis followed in some cases after antitoxine was given, and the kidney trouble was attributed to antitoxine, but no such trouble followed in any of our cases. The action of the antitoxine was remarkably uniform. As a rule, the temperature went up about one degree in the first twelve hours, but after fifteen to eighteen hours began to decline and went steadily towards normal, which was reached in about forty-eight hours from the time the medicine was given. At the end of forty-eight hours convalescence was well established, temperature normal, membrane beginning to separate, and from that time convalescence went on without any interruption. The stage of development at the time the antitoxine was given had marked influence on its effect; the earlier it was given the shorter the interval from the administration to the beginning of convalescence, and also the convalescence was shorter and recovery more rapid and complete. Although it does better to give it early in the disease, yet its effect when administered in fully developed cases was prompt and satisfactory; but its good effects were longer in becoming manifest and convalescence was more protracted. The usual dose was from 1,000 to 2,000 units of Behring, Parke, Davis & Co., manufacture. It was not necessary to administer the second dose in any of our cases. Locally, we used a spray of peroxide hydrogen and astringents, but in one case no treatment except antitoxine was given, and it seemed to do as well as the others and recovered as promptly. Locally, no abscess or irritation followed its injection. We first used an ordinary hypodermic syringe to administer the antitoxine, but afterward a Roux's antitoxine syringe. In three cases in which it was given for its immunizing effect, it apparently was effective. GEORGE HORINE, M.D.

*Americus, Ga., Jan. 16, 1896.*

